# Response: Commentary: Heart rate variability and self-control–A meta-analysis

**DOI:** 10.3389/fpsyg.2016.01070

**Published:** 2016-07-14

**Authors:** Daniela Zahn, Mario Wenzel, Thomas Kubiak

**Affiliations:** Health Psychology, Johannes Gutenberg-University MainzMainz, Germany

**Keywords:** vagal tone, heart rate variability, self-regulation, parasympathetic activity, self-control

We read the commentary on our meta-analysis with great interest and we greatly appreciate that the authors developed suggestions for further research on heart rate variability (HRV) and self-control. While we mostly support these suggestions, we believe that it is necessary to clarify some points raised with respect to our meta-analysis.

The first point brought up by the authors of the commentary is the focus of our meta-analysis on HRV instead of vagal tone and the inclusion of studies using the standard deviation of RR intervals (SDNN). They argue that only vagal tone and HRV metrics reflecting vagal tone are expected to be linked to self-control according to both the Neurovisceral Integration model (Thayer and Lane, 2000; Thayer et al., 2009) and Polyvagal theory (Porges, 2007). We agree with the authors that parasympathetic or vagal tone should be more strongly related to self-control according to both theories. However, we would like to emphasize that we only included studies using SDNN, if they also reported HF or RMSSD, two metrics reflecting vagal tone. We also performed a moderator analysis with respect to HRV metrics, which did not reveal a moderating effect: In fact, all three metrics revealed similar effect sizes (Zahn et al., 2016, see Table 4), but for SDNN the effect size was not significant, probably due to the small number of studies and effects referring to SDNN. In sum, while the inclusion of studies using SDNN could be criticized from a theoretical point of view, we carefully evaluated the impact of the inclusion of SDNN on the results of our meta-analysis and did not find any evidence for an impact on the overall relationship between HRV and self-control.

The second issue raised in the commentary refers to the fact that we only included studies on HRV at rest, thereby excluding studies on HRV/vagal reactivity. The authors of the commentary reason (1) that this is not clearly stated throughout our manuscript, which might lead to an overgeneralization of our findings, and (2) that HRV reactivity might be associated more strongly with self-control. However, we believe that we made the focus of the current meta-analysis on HRV at rest quite clear because we emphasized HRV at rest in the abstract and also explicitly mentioned the exclusion of studies on HRV reactivity as a limitation in the discussion section. We chose to refrain from the inclusion of studies on HRV reactivity because of the following reasons: First, while both trait HRV (which is normally assessed by a single measurement of HRV at rest before the tasks) and HRV reactivity might be affected by several situational factors, *different* situational factors may influence trait HRV, and HRV reactivity: For example, task characteristics such as level of movement required during the task may affect HRV reactivity more strongly than trait HRV. These task characteristics would then affect the overall relationship between HRV and self-control, although they are probably only relevant to the HRV reactivity-self-control connection. This would be very difficult to disentangle in a moderator analysis focusing on both trait HRV and HRV reactivity. Second, HRV reactivity plays a major role in Polyvagal theory but only a minor role in the Neurovisceral Integration model. Thus, we chose to focus only on the common assumptions of both theories, i.e., the assumptions referring to trait HRV. To conclude, we support the notion that further individual studies should examine both trait HRV and HRV reactivity. But we believe that a further meta-analysis focusing solely on HRV reactivity and self-control would be a more suitable approach than a meta-analysis integrating studies on both trait HRV and HRV reactivity.

The third issue addressed in the commentary refers to the inclusion of studies with a time gap between assessment of self-control and HRV measurement. The authors of the commentary point out that due to situational influences on HRV measurement these studies should have been excluded or their influence on the overall effect size should have been evaluated at least. We share the authors' opinion that HRV and self-control should be best assessed during the same session, but believe that it is necessary to clarify some aspects: First, our study quality criterion “Assessment of HRV and self-control at the same session” also referred to the *explicit reporting* of assessment of HRV and self-control performance at the same session. Three studies out of 26 failed to meet this criterion, with only one study actually reporting a time gap between both measurements. We repeated all analyses without this study and found no changes in the results except the already reported change in publication bias, which we have elaborated in great detail in the discussion section of our article (Zahn et al., 2016, p. 22). Finally, we would like to emphasize that we actually evaluated each single component of the study quality score as a possible moderator and reported the results (Zahn et al., 2016, p. 20): We did not find a significant moderating influence of the time gap between both measurements (Zahn et al., 2016, p. 20). In sum, while we agree that further research would benefit from an assessment of HRV and self-control at the same session to minimize situational influences, our results do not support the notion that the time gap between both measurements has as much impact on the results of the meta-analysis as assumed by the authors of the commentary.

To conclude, in our opinion, the points raised in context with our meta-analysis mostly reflect major issues of the research on HRV and self-control in general. To address these issues, we believe that more work is necessary both on a conceptual and on an empirical level. While further research is clearly needed with respect to the role of trait- and state-like aspects of HRV and self-control, we believe that it would be most helpful for future research to derive a consensus about terminology (e.g., HRV vs. parasympathetic activity/vagal tone), the HRV metrics used in the context of self-control as well as about the settings of HRV assessment.

## Author contributions

DZ wrote the first version of the commentary, MW and TK provided additional comments to improve it.

### Conflict of interest statement

The authors declare that the research was conducted in the absence of any commercial or financial relationships that could be construed as a potential conflict of interest.
